# Avoiding background knowledge: literature based discovery from important information

**DOI:** 10.1186/s12859-022-04892-8

**Published:** 2023-03-14

**Authors:** Judita Preiss

**Affiliations:** Information School, University of Sheffield, S1 4DP Sheffield, UK

**Keywords:** Literature based discovery, Subject–predicate–object triples, Machine learning, Timeslicing gold standard

## Abstract

**Background:**

Automatic literature based discovery attempts to uncover new knowledge by connecting existing facts: information extracted from existing publications in the form of $A \rightarrow B$ and $B \rightarrow C$ relations can be simply connected to deduce $A \rightarrow C$. However, using this approach, the quantity of proposed connections is often too vast to be useful. It can be reduced by using subject$\rightarrow$(predicate)$\rightarrow$object triples as the $A \rightarrow B$ relations, but too many proposed connections remain for manual verification.

**Results:**

Based on the hypothesis that only a small number of subject–predicate–object triples extracted from a publication represent the paper’s novel contribution(s), we explore using BERT embeddings to identify these before literature based discovery is performed utilizing only these, important, triples. While the method exploits the availability of full texts of publications in the CORD-19 dataset—making use of the fact that a novel contribution is likely to be mentioned in both an abstract and the body of a paper—to build a training set, the resulting tool can be applied to papers with only abstracts available. Candidate hidden knowledge pairs generated from unfiltered triples and those built from important triples only are compared using a variety of timeslicing gold standards.

**Conclusions:**

The quantity of proposed knowledge pairs is reduced by a factor of $10^3$, and we show that when the gold standard is designed to avoid rewarding background knowledge, the precision obtained increases up to a factor of 10. We argue that the gold standard needs to be carefully considered, and release as yet undiscovered candidate knowledge pairs based on important triples alongside this work.

**Supplementary Information:**

The online version contains supplementary material available at 10.1186/s12859-022-04892-8.

## Background

Literature based discovery (LBD) is an automatic technique for detecting as yet unobserved connections between information contained in different documents. With many applications in the biomedical domain, such as confirming suspected connections [1] or drug re-purposing [2], LBD is only limited by computational resources. Time limitations on researchers, on the other hand, dictate they specialize in relatively narrow domains, and thus potentially miss connections to publications out of their specialized area. However, generation of all inferable links from a large document collection can result in a vast quantity of candidate hidden knowledge pairs (CHKPs), making the result effectively unusable. Since many of the generated CHKPs represent effectively background knowledge, we propose using machine learning to restrict the information extracted from articles to their novel contributions and therefore reduce the amount of information passed to the LBD system. This not only decreases the quantity of CHKPs generated, but also—if a gold standard which avoids rewarding background knowledge CHKPs is used—the quality (measured by precision) increases.

The original approach to LBD assumed all term pairs co-occurring in an article’s title were related [3]. A previously unseen connection made using such relations, from a source term *A* to a target term *C* via one linking term *B* (the A–B–C model), was considered a CHKP. Following all such connections is termed open discovery. The approach was originally used in closed mode, where a relation was suspected between *A* and *C* and a connection was confirmed by extracting linking terms. Using LBD in closed discovery mode avoids the problem of generating vast quantities of CHKPs and reduces computational demands, but it is only applicable if *A* and *C* terms are known.

A number of approaches to reducing the quantity of CHKPs have been explored, with the most common filtering approaches including: (a) stop-listing, with stop-lists either created manually [4], from other resources [5], or automatically [6]; (b) restrictions of source or target concepts to certain Unified Medical Language System Metathesaurus (UMLS) [7] semantic types [8]; (c) filtering co-occurrence relations based on statistical significance tests [9]; (d) filtering relations using an automatic relation extraction system such as SemRep [10] and restricting the relations employed to a subset [11]. While it is possible to filter or re-rank the resulting CHKPs, in this work we focus on reducing the input to the LBD system. To this end, we integrate a novel approach based on embeddings of grammatical triples, designed to identify triples describing the novel contributions of publications, with an LBD system.

## Results and discussion

**Fig. 1:**
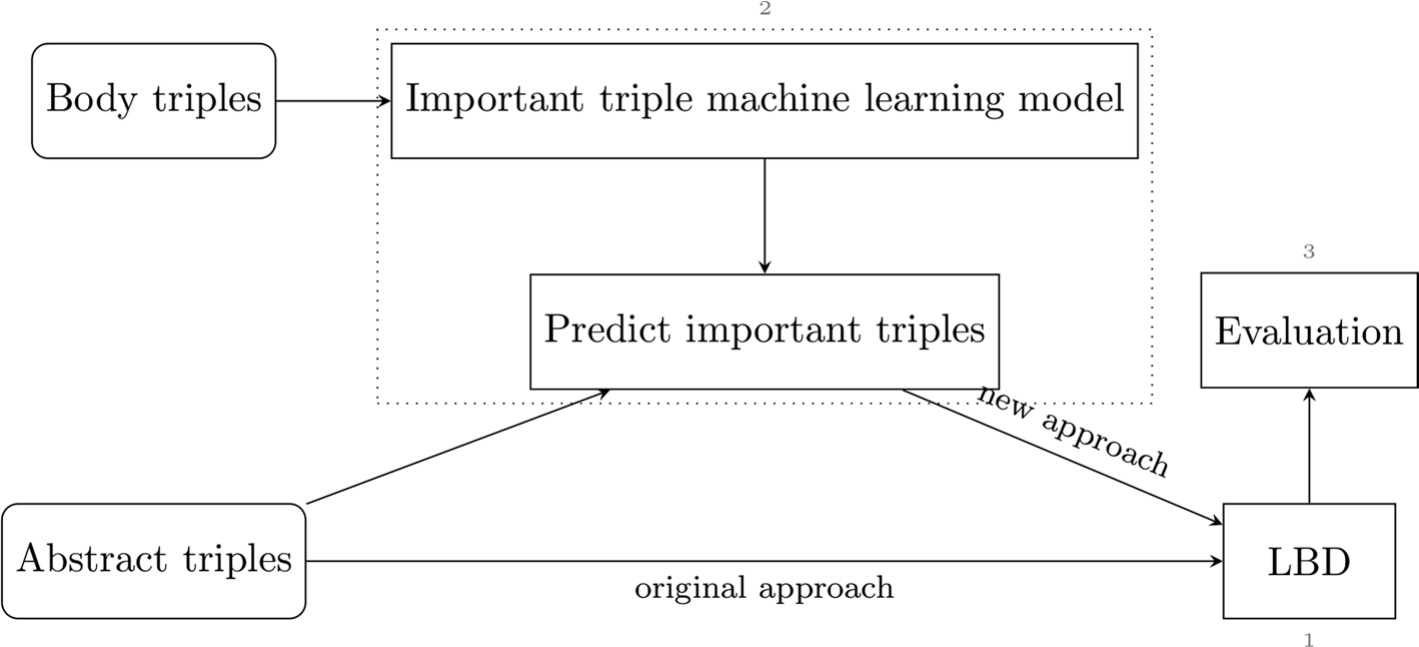
Overview of the main parts of the work

Three main parts of the work, which can be seen in Fig. 1, are implemented and evaluated: (1) the LBD system, (2) the machine learning (ML) model identifying important, novelty representing, subject-predicate-object (SPO) triples, and (3) the evaluation of the resulting CHKPs.

### Literature based discovery system

The A–B–C [3] LBD model, which proposes a CHKP $A-C$ for two previously unconnected terms *A* and *C* if relations between $A \rightarrow B$ and $B \rightarrow C$ exist, is clearly strongly dependent on the definition of a relation. When using UMLS, this LBD system is based on vertices and edges: the vertices correspond to UMLS concept unique identifiers (CUIs) and edges represent a relation between two CUIs. This suggests a graph structure and therefore the LBD system was implemented using python’s networkX library. Aside from giving some general information about the network (such as the average in or out degree), the library provides access to the (sparse) adjacency matrix, which can be efficiently squared. Since the square of an adjacency matrix $A^2= (s_{ij})$ has the property that $s_{ij}$ represents the number of walks of length two from vertex *i* to vertex *j*, any $s_{ij}$ in the square matrix which were zero in the original adjacency matrix represent one step CHKPs.

### Identification of important triples

Using SPO triples in a general A–B–C LBD system improves the quality of CHKPs [12], but it is not clear how much new (not commonly known) content such triples contain: they can represent specific information, such as *“imiquimod 50 MG/ML Topical Cream - TREATS - Erythema”* but they can also merely represent what can be termed background knowledge, such as *“Russian Federation - ISA - Countries”* or *“Diabetes - PROCESS_OF - Patient”*. The biomedical domain tuned SPO extraction tool, SemRep [10], produces such triples, outputting concepts mapped to UMLS CUIs (yielding *C0011847 PROCESS_OF C0030705* for the final example). Using UMLS CUIs reduces difficulties which would arise from failing to identify multi-words (e.g. *Russian Federation*) and word sense disambiguation (where a term can be used to refer to different concepts). However, many potential, unhelpful, CHKPs will still be generated from connections via e.g. *patient* in the triple above. To reduce the number of such generic triples, we build on recent work [13] to identify and use in LBD only those triples that represent a paper’s novel contribution via two steps: the creation of a BERT language model from SPO triples, which produces triple embeddings, and the utilization of the model in a downstream classification task.

#### BERT language model

Deep learning is used to map text into a vector space in a way that similar concepts are close under a geometric comparison. Such vector embeddings can pertain to individual words [14] or entire sentences (e.g. BERT [15]). We explore applying BERT’s masked language modeling (MLM) approach to SPO triples directly to produce SPO embeddings.

The large body of triples for training is drawn from the publicly available semantic MEDLINE Database (SemMedDB ver43_r) [16] which contains 107,385,842 SemRep predications extracted from all available MEDLINE citations. To learn a language model, each triple is treated as a three word sentence (since SemRep predications are between UMLS CUIs, rather than words / terms, they always have exactly three elements). BERT [15] is used to train the language model in combination with a word level tokenizer. This tokenizer avoids the creation of subword tokens which, since UMLS CUIs have the format C[0-9]{7} (e.g. C0012984), lack meaning. The MLM model then hides one of the components of the SPO triple and optimizes the prediction of the masked element based on its context. Hyperparameter tuning is performed on vocabulary size and the number of hidden layers.

#### Classification

In previous work [13], a feature based classifier was built based on the hypothesis that a novel contribution of a paper will usually appear (potentially rephrased) in both the abstract of a paper as well as the body. A notion of SPO triple similarity was defined to enable SPO triples appearing in the body of the paper to be annotated as ‘important’ (similar to a triple in the abstract) or ‘not’ (dissimilar to all triples in the abstract, and therefore unlikely to be describing a novel contribution of the paper). This training data, along with features drawn from a paper’s full text (such as the name of the section the triple appeared in, and the TextRank [17] of the triple’s sentence), enabled a classifier to be constructed which annotates SPO triples with a binary decision regarding each triple’s ‘importance’. However, the approach was limited to papers with available and extractable full text. An approach based on embeddings does not have such a requirement.

The BERT SPO language model is used in a classification downstream task to annotate each input SPO triple with a binary decision reflecting its ‘importance’. The training set for classification is created from the CORD-19 dataset [18] (2021-06-14) which contains extracted text from PDFs of 219,710 COVID-19 articles. SemRep (v1.8) SPO triples were extracted from the entire collection and the measure of similarity between triples used in [13] is employed. Specifically, for two triples, $cui_{11}-rel_1-cui_{12}$ and $cui_{21}-rel_2-cui_{22}$, the measure compares the subject CUIs ($cui_{11}$ with $cui_{21}$) and the object CUIs ($cui_{12}$ with $cui_{22}$) by computing the cosine similarity of their cui2vec embeddings [19], while the predicates ($r_1$ and $r_2$) are compared using cosine similarity of their GloVe embeddings [20]. Each cosine similarity comparison yields a maximum value of 1, with their sum giving similarity between two triples in the interval [0, 3]. The maximum similarity between an SPO triple within the body of an article (body triple) and all SPO triples in the abstract quantifies the body triple’s “importance”. A threshold on this similarity value can be used to create a (binary problem) SPO triple importance classification training set: with similarities above the threshold deemed important.

To ensure classifier training data did not overlap with test data (LBD is performed on CORD-19 publications before 2021-01-01), a 50,000 instance subset of 2021-01-01 to 2021-03-29 portion of the CORD-19 dataset was used to train the classifier. In addition, this training set is composed of SPO triples appearing in bodies of the corresponding articles, while LBD performed using the embedding model utilizes abstract SPO triples only. As in [13], the training instances in the chosen time period were selected such that the similarity value for ‘important’ triples is $\ge 2.5$ and ‘not important’ triples have a similarity value $\le 1.5$. The similarity gap was introduced in the above-mentioned previous work to ensure that the ‘important’ and ‘not important’ triples were sufficiently different not to cause confusion, and the thresholds were optimized (on a validation set).

The BERT model was compared with an optimized Keras [21] feature based model presented in [13]. The model is composed of fully connected layers of halving sizes with the final layer having size 1. The activation function used was ReLU [22] throughout except for the one node output layer which employs the sigmoid activation function, and the loss function was binary cross entropy. The best performing feature model had depth 4, trained for 200 epochs, had 0 dropout, randomly under-sampled the ‘not important’ data to create equal sized classes and used the accuracy metric in optimization. Since some features can occasionally have missing values, specifically the depth of the two CUIs involved in the triple and TextRank, separate models were trained for triples with missing features and the model which used the highest number of available features was selected for each SPO triple to be classified.

### Evaluation

The evaluation of CHKPs is inherently problematic as—by definition—there’s no gold standard for previously undiscovered connections. Two techniques are usually employed: (1) replication of existing discoveries, or (2) timeslicing [8]. The disadvantage of replication of existing discoveries is that the performance on the small number of discoveries available can be misleading—a system can either appear to have poor performance due to missing a single discovery or two, or appear to have excellent performance by suggesting a large number connections (most of which are not worthy of further pursuit). In timeslicing, CHKPs are generated from relations drawn from publications before a chosen date *D* and relations in publications after this date form the gold standard. When applied to a general LBD system, timeslicing can yield both precision and recall values, though the gold standard is clearly dependent on the quality of relations. Its larger scale, and therefore more representative results, makes timeslicing the chosen evaluation for this work. In this work, LBD is performed using publications which have full texts in the CORD-19 dataset. This allows three timeslicing evaluation gold standards to be explored:

1. SPO triples appearing in SemMedDB [16] (SemRep transformed abstracts of all PubMed articles, v43_r) publications published after the cut-off date *D*.
2. SPO triples appearing in abstracts of the CORD-19 dataset after the cut-off date *D*.
3. Important triples appearing in the CORD-19 dataset after the cut-off date *D*.

While LBD based on the CORD-19 dataset may be more likely to concern COVID-19 (gold standard ), out of domain connections are expected to reach further (gold standard ). However, gold standard  allows the investigation of performance with a decreased danger of rewarding background knowledge triples.

Since this employs only important triples to perform LBD, i.e. a subset of the original full set of triples extracted from the document collection, the reduction in quantity of triples needs to be explored. There are 1,999,545 distinct SPO triples within the full body texts, with 189,158 of these appearing in the abstracts. Using the best performing feature based model to extract SPO triples which are deemed important reduces the set of SPO triples to 6964 (of which 1223 appear directly in the abstract) before the cut-off date of 2021-01-01 from 153,057 documents. The fine-tuned BERT model annotates 10,046 triples as important. Assuming each document presents exactly one novel contribution which can be extracted as an SPO triple, 153,057 important triples would be expected. Since both approaches produce significantly fewer triples, either the assumption that novel contributions can be extracted as SPO triples is incorrect, or the filtering performed by the models is too aggressive. However it should be noted that for closed LBD other approaches can be used to verify a suspected connection, and for open LBD (where all inferable connections are sought) completeness is not the goal—the quantity of generated CHKPs needs to allow for manual inspection and therefore missing some inferable connections to yield high confidence CHKPs is acceptable.

Indeed, as shown in Table 1, the quantity of total CHKPs generated from the BERT important triples is almost a factor of $10^3$ lower than the CHKPs generated from all abstract triples. Given the sheer number of CHKPs generated from triples taken from abstracts, it is clear that reporting recall—a measure of completeness—does not make sense: producing all possible new connections would give 100% recall, however, the usefulness (whether it is possible for an expert to manually select connections worthy of further exploration) of such a system would be negligible. This measure of usefulness is partly represented by precision, which quantifies the percentage of CHKPs that were found in the gold standard and therefore takes into account the quantity of CHKPs returned. However, whether abstract triple or important triple based LBD performs better varies with the gold standard: abstract triple version performs better on the abstract triple based entire PubMed gold standard, while the important triple based LBD outperforms the original version on the two gold standards generated from CORD-19 data (the best performing triples source is highlighted in bold for each gold standard).

While this may appear surprising since *PubMed abstract* and *CORD-19 abstract* gold standards are both composed of SPO triples appearing in abstracts, we hypothesize that the smaller size (see Table 2 for sizes of the three gold standards) reduces the quantity—and therefore the reward—given to the more general, background knowledge, CHKPs. This hypothesis is consistent with the performance on the last gold standard, which is created from important triples (as deemed by the machine learning algorithm) appearing in the CORD-19 dataset after the cut-off date. Assuming the machine learning algorithm is correctly discarding background information triples, connections such as *at home—cancer patient* (made via *complication* and *disease*) will neither appear in the *CORD-19 important* gold standard nor the output of the important triple LBD approach. This connection is, however, produced by the original LBD approach and is rewarded against the *PubMed abstract* gold standard. This suggests that a general timeslicing gold standard may be presenting misleading results when used for the evaluation of LBD systems due to its propensity to reward background knowledge.

**Table 1:** Percentage precision of LBD using all abstract triples versus BERT important triples against three gold standards

			Percentage precision	
Triples source	Total CHKPs	PubMed abstract	CORD-19 abstract	CORD-19 important
Abstract	11,736,755	**0.777**	0.183	0.004
Important	12,512	0.408	**0.304**	**0.040**

**Table 2:** Gold standard sizes

	PubMed abstract triples	CORD-19 abstract triples	CORD-19 important triples
Size	1,693,799	148,036	4158

## Conclusions

To address the problem of LBD generating a large number of CHKPs which effectively encode background knowledge, we propose employing a machine learning algorithm to identify important relations before LBD. The idea of LBD is to make inferences from novel discoveries in publications, and the identification of important relations before LBD allows for a reduction in relations to just this set. To this end, a classifier built on top of BERT triple based language model is trained using a training set automatically created from CORD-19. When the classifier is employed to reduce the set of triples, the quantity of CHKPs was found to reduce by a factor of $10^3$.

The CORD-19 dataset is also employed to investigate the hypothesis that the direct use of timeslicing for the evaluation of a general LBD system may be rewarding background knowledge and thus may be presenting slightly misleading results. Using gold standards with a lower quantity of background knowledge boosts the performance of the importance based LBD. CHKPs proposed by the importance based LBD that remain after all CHKPs appearing in any of the three gold standard are removed are released alongside this work.

## Methods

The work aims to reduce the quantity of CHKPs generated by open discovery LBD, white retaining or improving their quality, by hypothesizing that novel contributions of a publication can be identified using language modeling from SPO triples.

The work consists of three main parts: (1) the construction of an A–B–C LBD system, (2) the creation of a fine-tuned BERT model identifying important, novelty representing, SPO triples in publications based on a language model trained on SPO triples, and (3) the evaluation of the resulting CHKPs using timeslicing. A number of different timeslicing based gold standards are discussed.

Precision values are calculated when using three different gold standards showing a reduction in CHKPs by a factor of $10^3$, and highlighting the importance of selecting a gold standard which emphasizes important knowledge.

## Supplementary Information

**Additional file 1. **CHKPs proposed by the importance based LBD that remain after all CHKPs appearing in any of the three gold standard are removed.

## Data Availability

The CHKPs generated from ‘important’ relations identified in the 2021-06-14 release of the CORD-19 data (available from https://www.semanticscholar.org/cord19/download) are included in Additional file 1.

## Abbreviations

COVID-19: Disease caused by SARS-CoV-2, the coronavirus that emerged in December 2019
CORD-19: The COVID-19 open research dataset
CHKP: Candidate hidden knowledge pair
CUI: The concept unique identifier within UMLS
LBD: Literature based discovery
ML: Machine learning
SPO: Subject-predicate-object
UMLS: Unified medical language system
